# Editorial: LGBTQ Parents and Their Children During the Family Life Cycle

**DOI:** 10.3389/fpsyg.2021.643647

**Published:** 2021-02-18

**Authors:** Nicola Carone, Henny M. W. Bos, Geva Shenkman, Fiona Tasker

**Affiliations:** ^1^Department of Brain and Behavioral Sciences, University of Pavia, Pavia, Italy; ^2^Research Institute of Child Development and Education, University of Amsterdam, Amsterdam, Netherlands; ^3^Interdisciplinary Center Herzliya (IDC), Hertzlya, Israel; ^4^Department of Psychological Sciences, School of Science, Birkbeck University of London, London, United Kingdom

**Keywords:** LGBTQ parent, child development, family relationships, assisted reproduction, adoption

Over the past few decades the number of children growing up in LGBTQ-parent families has increased dramatically within the context of shifting sociopolitical and legal climates around the world, more favorable attitudes toward diverse family forms, and expanded access to assisted reproduction technology and adoption (Goldberg et al., 2018). Among diverse LGBTQ-parent family forms, lesbian and gay stepfamily arrangements formed post heterosexual relationship (PHR) dissolution likely represent the most common formation (Tasker and Lavender-Stott, 2020). Contrary to prevailing expectations, early studies with mothers who came out as lesbians showed that they were just as likely to have good mental health and positive relationships with their children as were heterosexual mothers, and that their children were no more likely to show emotional and behavioral difficulties, poor performance at school, or atypical gender role behavior than were children with heterosexual parents (Tasker, 2010; Patterson, 2017).

Along with research on lesbian stepfamily arrangements, what we currently know about parenting and the adjustment of children whose parents are a sexual and/or a gender minority is still mainly limited to lesbian-parent families through donor insemination (Bos and Gartrell, 2020). Planned lesbian-parent families were also created by adoption (Farr et al., 2020), by sexual intercourse with a man who would not be a father to the child and by elective co-parenting, whereby the mother had a child with a man who was not her partner but played a role in raising the child (Jadva et al., 2015). The rapid increase in openly lesbian women having children at that time became known as “the lesbian baby boom” (Patterson, 2017).

Studies with lesbian-parent families formed through donor insemination confirmed the positive outcomes found for lesbian stepfamily arrangements. In addition, studies increasingly supplemented a between-difference approach (in which planned lesbian-parent families with donor-conceived offspring were compared with heterosexual-parent families) with a within-difference approach, thus shedding light on the nuanced family dynamics and unique family processes specific to lesbian parents and their donor-conceived offspring (e.g., relationships with donors, parenting with different biological relationships to the child) (Gato, 2016; Bos and Gartrell, 2020). For instance, crucial insights have been generated by the U.S. National Longitudinal Lesbian Family Study (NLLFS), the first study to have examined the experiences and outcomes of donor-conceived offspring and their lesbian parents from conception to mid-adulthood (Gartrell, 2021), emphasizing both the adverse effect of stigmatization on child development over time (Bos et al., 2021) and the absence of difference in psychological adjustment among offspring with an anonymous, a known, or an open-identity donor (Bos and Gartrell, 2011; Carone et al., 2021).

In the last two decades, some longitudinal studies have been conducted with adoptive lesbian- and gay-parent families (e.g., Goldberg and Garcia, 2016; Farr, 2017; McConnachie et al., 2020), confirming that the quality of family processes and the stigmatization occuring in the outside world are more relevant to child adjustment than family structure. Also preliminary cross-sectional evidence is now available on the family life dynamics and the positive adjustment of children born to surrogacy and raised in a two-father (e.g., Carone et al., 2018b, 2020b; Golombok et al., 2018; Green et al., 2019; Berkowitz, 2020) or a gay single father (Carone et al., 2020a) family, as well as on the challenges faced and unique strengths among school-age children, adolescents, and emerging adults raised in sexual minority-parent families (Kuvalanka and Goldberg, 2009; Tasker and Granville, 2011; Gartrell et al., 2012; Kuvalanka et al., 2014; Farr et al., 2016a; Koh et al., 2020). Increasingly, there is also a growing interest in studying the experiences and outcomes of bisexual mothers (Tasker and Delvoye, 2015), and transgender or non-binary parents (Kuvalanka et al., 2018; Carone et al., 2020c).

To date, considerable insights have been gained into many aspects of LGBTQ family life, benefitting from theoretical advances (Farr et al., 2017; Prendergast and MacPhee, 2018) and increased methodological rigor, including the use of nationally representative and large data sets (e.g., Bos et al., 2016; Riskind and Tornello, 2017; Calzo et al., 2019), longitudinal designs (e.g., Goldberg and Garcia, 2016; Farr, 2017; McConnachie et al., 2020; Gartrell, 2021), multiple informants (e.g., Farr, 2017; Carone et al., 2018b; Golombok et al., 2018; Simon and Farr, 2020), mixed-method designs (e.g., Farr et al., 2016b; Simon and Farr, 2020), and meta-analyses (Fedewa et al., 2015; Miller et al., 2017). Notwithstanding, research conducted thus far has been limited in terms of the predominant populations studied (e.g., lesbian parents, middle class, White families) and topics under investigation (e.g., child behavioral adjustment, parenting quality). Also, the proliferation of studies in diverse international contexts outside of the U.S. (e.g., Australia, Canada, Italy, the Netherlands, and the U.K.) has not seen the same interest for LGBTQ-parent families living in non-Western contexts [for exceptions, see Erez and Shenkman (2016), Shenkman and Shmotkin (2019), and Shenkman (2020)]. Thus it remains to be seen how combinations of specific cultural and socio-demographic aspects (e.g., parents' class and socioeconomic status, education level, and both parents, and children's race/ethnic background) may shape individual, couple, and family experiences and outcomes (Costa and Shenkman, 2020).

To this end, the current Research Topic brought together experts in the field from different socio-cultural settings around the world (i.e., Chile, France, Israel, Italy, the Netherlands, New Zealand, Portugal, Sweden, the U.K. and the U.S.) to focus on different aspects of the experiences and outcomes of LGBTQ parents and their children, throughout their family life cycle. A total of 14 articles are contained within our Research Topic on diverse family forms of LGBTQ parents using various methodological approaches and including qualitative research and quantitative studies involving between-difference comparisons and within-difference contextual detail. We have also been able to include papers on a diverse array of LGBTQ-parent family forms covering 8 substantive areas: (1) intentions and desire to become a parent among lesbian women (van Houten et al.), lesbian, gay, and bisexual young adults (Gato et al.; Tate and Patterson), transgender and non-binary people (Tasker and Gato); (2) perceptions of the most challenging and most optimal experiences of raising children in non-traditional families among the first generation of lesbian parents through donor insemination (Gartrell et al.); (3) consideration of the legal restrictions experienced by lesbian parents as remembered by young adult offspring (Malmquist et al.); (4) associations between division of labor and parental, couple, and child outcomes among transgender and gender-non binary parents (Tornello), and lesbian and gay parents through assisted reproduction (Van Rijn—Van Gelderen et al.); (5) stigmatization and contextual influences upon parenting and psychological adjustment among adoptive lesbian and gay parents (Farr and Vázquez; Goldberg and Garcia); (6) longitudinal associations between children's experiences of their surrogacy origins in gay-parent families and family discussions about conception within the context of attachment security (Carone et al.); (7) perceptions of lawyers and social workers toward adopted children with lesbian and gay parents (Scherman et al.) and finally (8) explorations of pathways to parenthood in non-Western contexts considering data on the psychological well-being of Israeli gay parents through surrogacy (Shenkman et al.) and the family lives of Chilean lesbian parents in the context of a heteronormative and Christian society (Figueroa and Tasker).

The 14 articles included in this Research Topic provided a comprehensive contemporary picture depicting the realities and experiences of members of LGBTQ-parent families. In a similar vein, these papers also invite additional questions, particularly from a longitudinal, multi-informant, contextual, and intersectional perspective. Specifically, given the different regulations governing same-sex marriage around the world, future research questions may relate to how (not) gaining marriage equality affects families, especially regarding relationship commitment, divorce, societal stigma, and children's relationship quality with biological and non-biological (legal and non-legal) parents, grandparents, uncles, and aunts. Also, diversity in both family composition and pathways into parenthood means that parents need to explain or contextualize this for their children. In this respect, the little that is currently known about parents' socialization practices and strategies surrounding family structure is largely limited to lesbian and gay adoptive parent families (Goldberg et al., 2016; Wyman Battalen et al., 2019). How parents socialize their children to family diversity in other sexual and/or gender minority-parent families still remains to be addressed. In a similar vein, more first-hand accounts are needed of children's views on their family form (Gartrell et al., 2012; Zadeh et al., 2019), as well as on their understanding of origins and contact experiences with birth parents, gamete donors, and/or surrogates (Blake et al., 2016; Carone et al., 2018a; Farr et al., 2018; Koh et al., 2020).

Beyond gender, sexual orientation, number of parents, and pathway to parenthood, LGBTQ parent-families may also differ on a number of socio-demographic and health aspects, including race/ethnicity, social class, physical well-being, and geography (e.g., nationality, living in an urban or rural area). In this vein, much more work from an intersectional perspective is needed to understand the lives of minority, binational, and immigrant LGBTQ-parent families with regards to the complex juxtaposition of multiple minority stressors (e.g., racism, heterosexism, cisgenderism, disability status, lack of resource availability). Finally, although included in the panoply of papers on LGBTQ parenting addressed in this Research Topic, it is paramount to note that bisexual, queer, and trans-/gender diverse-parent families remain understudied family forms.

The ways in which LGBTQ-parent family arrangements can be built will continue to evolve in the near future. In this vein, in 2010 lesbian couples began to have children through shared biological motherhood, where one partner provides her eggs that will be fertilized with donor sperm and the other partner carries the pregnancy; the resulting children will have a genetic mother and a gestational mother (Marina et al., 2010). Furthermore, it may be just a matter of time and legislative endeavor until the possibility of using gametes derived from human embryonic stem cells will enable both partners in same-sex couples to be genetically related to their child (Adashi and Cohen, 2020). How children will develop and how family processes will be articulated in these upcoming LGBTQ-parent families are still to be seen. Notwithstanding, what contemporary research has clearly demonstrated is that although the family structure does not affect the development of children with sexual and/or gender minority parents, discrimination and stigmatization against their family does.

## Author Contributions

All authors listed have made a substantial, direct and intellectual contribution to the work, and approved it for publication.

## Conflict of Interest

The authors declare that the research was conducted in the absence of any commercial or financial relationships that could be construed as a potential conflict of interest.
